# Linking stormwater Best Management Practices to social factors in two suburban watersheds

**DOI:** 10.1371/journal.pone.0202638

**Published:** 2018-08-23

**Authors:** P. Kanoko Maeda, Victoria Chanse, Amanda Rockler, Hubert Montas, Adel Shirmohammadi, Sacoby Wilson, Paul T. Leisnham

**Affiliations:** 1 Department of Environmental Science and Technology, University of Maryland, College Park, Maryland, United States of America; 2 Department of Plant Science & Landscape Architecture, University of Maryland, College Park, Maryland, United States of America; 3 Maryland Sea Grant Extension, University of Maryland, College Park, Maryland, United States of America; 4 Fischell Department of Bioengineering, University of Maryland, College Park, Maryland, United States of America; 5 School of Public Health, University of Maryland, College Park, Maryland, United States of America; University of Vermont, UNITED STATES

## Abstract

To reduce nutrient pollution in urban watersheds, residents need to voluntarily practice a range of stormwater Best Management Practices (BMPs). However, little is known about the underlying social factors that may act as barriers to BMP implementation. The overall goal of this study was to better understand barriers to BMP implementation by exploring the links among resident demographics, knowledge, and behaviors so that appropriate education can be more effectively developed and targeted. In 2014–2015, a detailed questionnaire was administered door-to-door to 299 randomly selected households in two sub-watersheds of the Chesapeake Bay basin to test relationships among resident demographics, knowledge and attitudes towards water resources and BMPs, and BMP implementation. Multifactor regression models showed that respondents who had greater knowledge of water resources and BMPs lived in households that implemented greater numbers of BMPs. In turn, resident BMP knowledge, or familiarity with BMPs, strongly varied with race and ownership status, with respondents who identified as Caucasian or within a collection of ‘Other’ races, and who were home owners, having greater BMP knowledge than respondents who identified as African American and who were home renters, respectively. Renters and members of homeowner’s associations were also less likely to implement BMPs independent of knowledge, possibly reflecting perceived or real bureaucratic or procedural barriers to good stormwater management. Overall, respondents preferred to receive educational materials on stormwater via pamphlets and YouTube videos. These results suggest that resident ownership status knowledge is important to determining the number of household BMPs, and that education outreach should probably target African American and renting households that have lower BMP knowledge and landlords and administrators of homeowner’s associations using well-planned print and video educational media.

## Introduction

Over half of the United States’ tributaries are designated as impaired by the US-EPA [1]. A major cause of this impairment is nonpoint source (NPS) pollution from stormwater runoff, which is defined as rainfall from diffuse locations that has not been taken up by other hydrologic pathways [2]. Agriculture is the leading source of NPS pollution in the United States [3], but the built environment is also a major and growing contributor [2]. Urban and suburban development usually reduce pervious surfaces, creating dramatic changes in the hydrologic regimes of whole watersheds [4]. For example, impervious surfaces reduce local infiltration rates and groundwater percolation, resulting in higher surface water runoff, peak discharges, and the export of sediment and associated nutrients (e.g., nitrogen, phosphorous) into local tributaries [4]. Moreover, urban greenspaces (e.g., gardens and lawns) often export excess nutrients, and when combined with higher stormwater runoff, promote eutrophication, while fertilizers, herbicides, insecticides, bacteria, and metals common in urban areas also pose additional risks to aquatic ecosystems [5].

To better manage the quantity and quality of urban stormwater, multiple legislative approaches through the 1972 Clean Water Act and later legislative amendments (e.g., 1987 Water Quality Act) have allowed more effective monitoring, policy development, and regulation of discharges [6]. Nevertheless, critics argue that improvements in urban stormwater management have been costly and incremental because this legislative approach has been ‘top-down’ and largely ineffective at regulating stormwater runoff from privately-owned households [7]. Because urban watersheds usually consist of numerous privately-owned residential parcels, household-scale Best Management Practices (BMPs), which are also often called stormwater control measures (SCM) (e.g., rain barrels, disconnected gutters, fertilizer reduction) are vital to help reduce urban stormwater and improve watershed quality [8–10].

Resource management theory and empirical research have shown that a myriad of economic, cultural, and other social factors (hereafter referred in combination as “socio-economic factors”) can affect how humans perceive their environment, and whether or not they implement particular conservation or management practices [11, 12]. Recent research suggests that urban stormwater management is limited by prioritizing technical solutions to reduce nutrient pollution, while not adequately incorporating socioeconomic factors into planning and decision-making [13]. For example, recent questionnaires indicate that flooding, safety, trash, and aesthetics are important stormwater-related concerns of most Americans, yet these concerns are typically not mitigated by common BMPs that instead aim to meet local and state regulatory discharge requirements (e.g., [7]). More effective urban stormwater management needs to engage residential communities in ‘bottom-up’ outreach interventions that promote the benefits of household BMPs and their implementation [14].

The Chesapeake Bay is arguably the United States’ most iconic estuary with a greater watershed area of over 64,000 mi^2^ (166,0000 km^2^) that intersects six states and the District of Columbia. Chesapeake Bay’s natural resources (e.g., seafood, recreational boating) provide over $678 billion in economic activity to its neighboring states and is considered a national treasure [15]. Chesapeake Bay is also emblematic of the rapid urbanization and degraded water quality that is observed across many watersheds in the United States and worldwide. Between 1950–1980, it is estimated that the Chesapeake Bay watershed lost 2.7 million acres of natural habitat to development, compared to 1.7 million acres from 1600–1950 [16]. As a result, urban stormwater runoff is the fastest growing source of pollution in the Chesapeake Bay watershed [17], contributing in 2015 an estimated 16%, 18%, and 24% of total nitrogen, phosphorous and sediment pollution, respectively [17]. Nitrogen and phosphorous from built environments have been shown to be major contributors to eutrophication in many parts of the Chesapeake Bay watershed [18–20] and pose severe risks to ecosystem and human health through algal blooms and hypoxic conditions that lead to fish kills and biodiversity loss [21]. In 2009, President Barack Obama enacted Executive Order 13508 to renew efforts to protect Chesapeake Bay [22]. One of the key strategies of this Order was to promote the research of socio-economic factors in watershed management [23]. Although effective management of urban stormwater likely depends on the knowledge and behavior of residents, there is a paucity of information of these factors in North America. Most studies that have investigated relationships between social factors and stormwater management have focused on qualitative analyses for planning or management purposes [24, 25] or public health interventions in developing countries [24, 26, 27]. Of the few quantitative studies on urban stormwater and social factors, their focus has been limited in demographic scope [14, 28, 29]. To better engage residential communities in ‘bottom-up’ outreach interventions and promote the implementation of household BMPs we need a better understanding of the complex relationships between the socio-economic demographics, knowledge and behaviors residents and their communities.

The main goal of this study is to examine resident knowledge, attitudes, and BMP practice along socio-economic and other demographic gradients. We administered a Knowledge, Attitude and Practice (KAP) questionnaire to socio-economically diverse households in two sub-watersheds in the wider Chesapeake Bay watershed, Wilde Lake watershed in the city of Columbia, Maryland and the Watts Branch watershed that spans the southeast portion of the District of Columbia and into neighboring Prince Georges County, Maryland. KAP surveys are often descriptive but many have been highly effective at finding statistically significant determinants of knowledge, attitudes and practices [29–32], and have been applied to a range of environmental contexts, including measuring baseline information on urban mosquito ecology and health impacts [33, 34], testing effects of education materials [35], and building awareness around drinking water quality [26, 27].

## Methods

### Study sites

Wilde Lake (WL; lat: 39.225°, lon: 76.867°) and Watts Branch (WB; lat: 38.891, lon: 76.928) watersheds are located more than 100 miles (160 km) upstream of the Chesapeake Bay along the flow paths of the Patuxent River and Anacostia Rivers, respectively. Both watersheds are suburban, cover areas of similar size (WL: 4.92 km^2^; WB: 9.24 km^2^), have a similar percentage of impervious surface (WL: 1.60, 32.5%; WB: 2.92 km^2^, 32.0%), and have severely impaired waters [36]. WL watershed occupies part of the Village of Wilde Lake in the City of Columbia, Maryland. Village governance is overseen by the Columbia Housing Association [37]. WL watershed has a highly educated, high-income citizenry and low minority population compared to national averages [38]. In contrast, WB watershed has a lower educated low-income citizenry with a high minority population compared to national averages, and no overarching housing association at a village or city level [38].

### Questionnaire

In 2014–2015, a detailed KAP questionnaire was administered to 299 randomly-selected households in WL and WB that were stratified by watershed [39]. The questionnaire was pretested to ensure the questions made sense to individuals within the target populations and then deployed door-to-door to one adult (>18 years-old) at each household. Only households in single-family detached/stand-alone residences with yards were visited by investigators and included in the study. Human subjects approval was obtained from the University of Maryland College Park Institutional Review Board (Protocol 413908–1). Oral consent was obtained from participants after investigators read a verbal script of the research and provided a copy and documented with a completed questionnaire. Oral rather than written consent was obtained because: 1) the research presented no more than minimal risk of harm to subjects; 2) the research involved no procedures for which written consent is normally required outside the research context; and 3) oral communication was deemed easier for participants to understand the study and to provide consent. The oral consent procedure was approved by the University Maryland College Park Institutional Review Board (Protocol # 11–0192). All data were analyzed anonymously.

### KAP data

Demographic information was collected on respondent age (18–49 years, >50 years), gender (male, female), race (Caucasian, African American, other), education (high school, college, graduate/professional), financial responsibility in the household (0, 25, 50, 75, or 100%), household income (<$75,000, >$75,000), household ownership status (rent, own), household association membership (Yes, No), and if children were present in the household (yes, no) (S1 Dataset). All demographic questions required respondents to select answers from a list. Questions on race, education, household income, ownership status had items that were then collapsed into the broader categories described above, which reflected natural breakpoints in the data and gave sufficient respondent numbers for statistical analyses (see below). Although every household in the WL watershed is a member of the Columbia Housing Association, 27.5% (28/102) of respondents from that watershed reported that they were not a part of a housing association. We decided to re-code these households as being a part of a housing association to increase the accuracy of this variable. As best as we could determine, all respondents (n = 190) in the Watts Branch watershed correctly reported membership in a housing association (n = 51, 26.8%). Membership in a housing association was difficult for us to confirm and is open to some variability in respondent understanding. Therefore, some caution is needed in interpreting the results of this question. Questionnaire responses personally relevant to the individual respondent were assumed to be representative of the household.

Respondents were assessed on two aspects of their knowledge and three aspects of their attitudes towards water resources and BMPs, and on their household implementation of BMPs. Respondents were assigned an overall water knowledge score based on their answers to eight questions on water resources and BMPs. Knowledge questions tested whether respondents could identify the watershed in which they lived, knew that stormwater is chemically untreated before being released into Chesapeake Bay, were aware of BMP rebate schemes and incentive programs, were aware of stormwater fees and how these fees were assessed, knew that nitrogen and phosphorous were mainly responsible for polluting Chesapeake Bay, and that the amount and cleanliness of stormwater is important to stream health. All questions required respondents to select answers from a list, and responses were coded correct or incorrect based on the respondent’s selection. For some questions, there was more than one correct answer. Correct answers were summed to yield an overall water knowledge score of 0–8. Respondents were also assigned a score for their knowledge of BMPs. Respondents were requested to indicate their familiarity with each of nine common household BMPs. Questionnaire pretesting revealed little confusion of BMPs with the names that we used to identify the practices in the questionnaire, and only a few study respondents requested that investigators further clarify at least one BMP. Responses indicating familiarity were summed across all nine BMPs to yield an BMP knowledge score from 0–9. Respondents were assessed on their attitudes toward water resources based on their agreement with six statements on a four-point (1, strongly disagree to 4, strongly agree) scale. The statements represented positive associations with and utilization of local and regional water resources and a perceived ability to help restore Chesapeake Bay. Respondents received a mean score of 1–4 as an overall index of their motivation to protect water resources, with a larger number indicating higher motivation. Respondents were assessed on their attitudes toward BMPs based on their selection of four negative and three positive statements for each of nine BMPs. The perception of each BMP was assessed from -4 to 3 and the mean score of all nine BMPs was calculated. Respondents were assessed on the degree to which they thought government versus individuals are responsible for stormwater runoff based on a 5-point Likert scale, with a larger number indicating greater responsibility for the individual. Respondents were asked a yes/no question about whether their household implemented any of nine household BMPs.

### Specific incentives, barriers and education

In addition to gathering information on demographics, knowledge, attitudes, and BMP implementation, we asked if statements of specific lifestyle preferences or concerns related to water resources applied to respondents. Lifestyle preferences included whether or not respondents like to garden, were member of a local watershed organization, volunteer at environmental events, enjoy fishing and crabbing, or consider themselves an environmentalist. Concerns included the health of the Chesapeake Bay, mosquito breeding in BMPs, or safety issues related to BMPs. Additionally, respondents were asked to indicate their preferred education/outreach approach from a list of options, including pamphlets, a local watershed training, or YouTube videos.

### Data analysis

**Fig 1:**
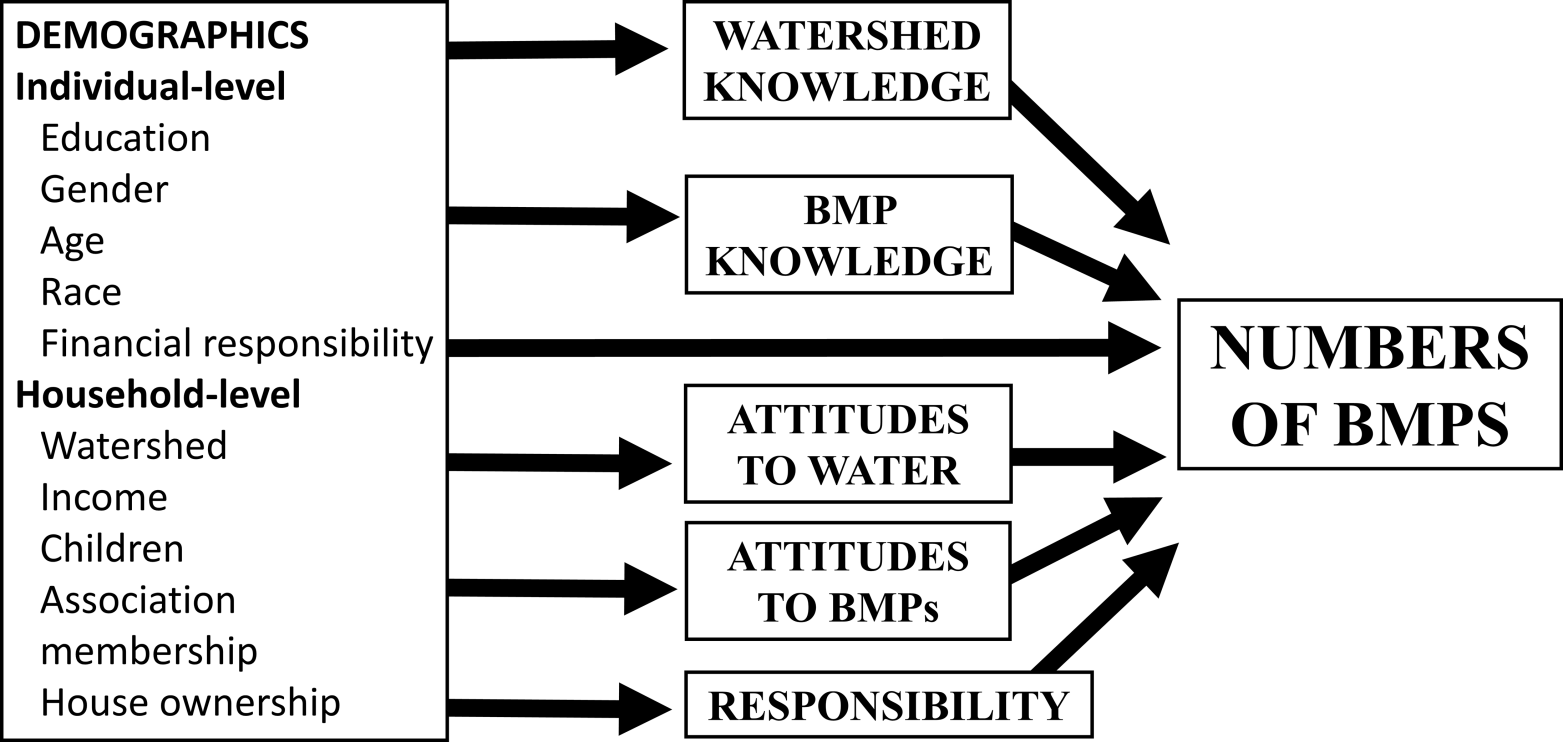
Conceptual diagram of the project’s step-wise analytical approach.

A total of 299 KAP questionnaires were administered with 205–299 responses for individual questions (S1 Dataset). Relationships among demographic, knowledge, attitudes, and BMP implementation variables were analyzed using generalized linear models following a step-wise approach [40] (Fig 1).

For each analysis, the appropriate error structure was chosen as described below. Overall water knowledge followed a Poisson error distribution. Knowledge of BMPs, and attitudes toward responsibility, water resources and BMPs were all normally distributed. Numbers of implemented BMPs at each household followed a negative binomial distribution. Household BMP implementation, or the implementation of at least one BMP, was treated as a binomial variable (presence/absence). In addition to household BMP implementation, separate analyses were undertaken to test predictors of the three most common individual BMPS: reducing fertilizer use, downspout disconnections and natural landscaping (see results). For each of these BMPs, knowledge and attitudes of the individual BMP were tested in addition to the overall water knowledge and attitudes of BMPs. Household BMP implementation and the implementation of the individual BMPs were treated as binomial variables (yes/no). Although BMP implementation is a household-level practice, individual-level demographic variables were still tested against it because they may either be representative of the household or affect respondent interpretation or self-reporting accuracy. For all analyses, factors with a screening significance of p<0.250 in single-factor tests were included in multi-factor models with all estimable two-way interactions. Final multi-factor models were selected using backward selection. First, all two-way interactions were non-significant and were eliminated. Then, if there was no significant loss of fit as evaluated by comparing AICc and -2 log-likelihood values, the next least significant factor was removed until all non-significant factors were removed or the model lost significant information compared with the previous model. Because a respondent's attitude on a specific BMP was not recorded if that respondent reported not having knowledge of the BMP, we ran separate sets of models that included either knowledge or attitudes toward specific BMPs. Multicollinearity was tested for all multi-factor tests by means of Variance Inflation Characteristics (VIF), with a VIF above 5 for variable indicating a problem [41]; but no VIF above 3.5 was detected. Incidence rate ratios (IRR) were obtained for significant factors in final models by using a modified Poisson approach with robust error variances [42]. We conducted chi-squared tests of association between respondent agreement to statements of specific lifestyle preferences and concerns with implementation of reducing fertilizer use, downspout disconnections and natural landscaping, with sequential Bonferroni correction for 24 tests. All tests used experimentwise α = 0.05; marginal significance was defined at α = 0.05–0.10.

## Results

### Knowledge

**Fig 2:**
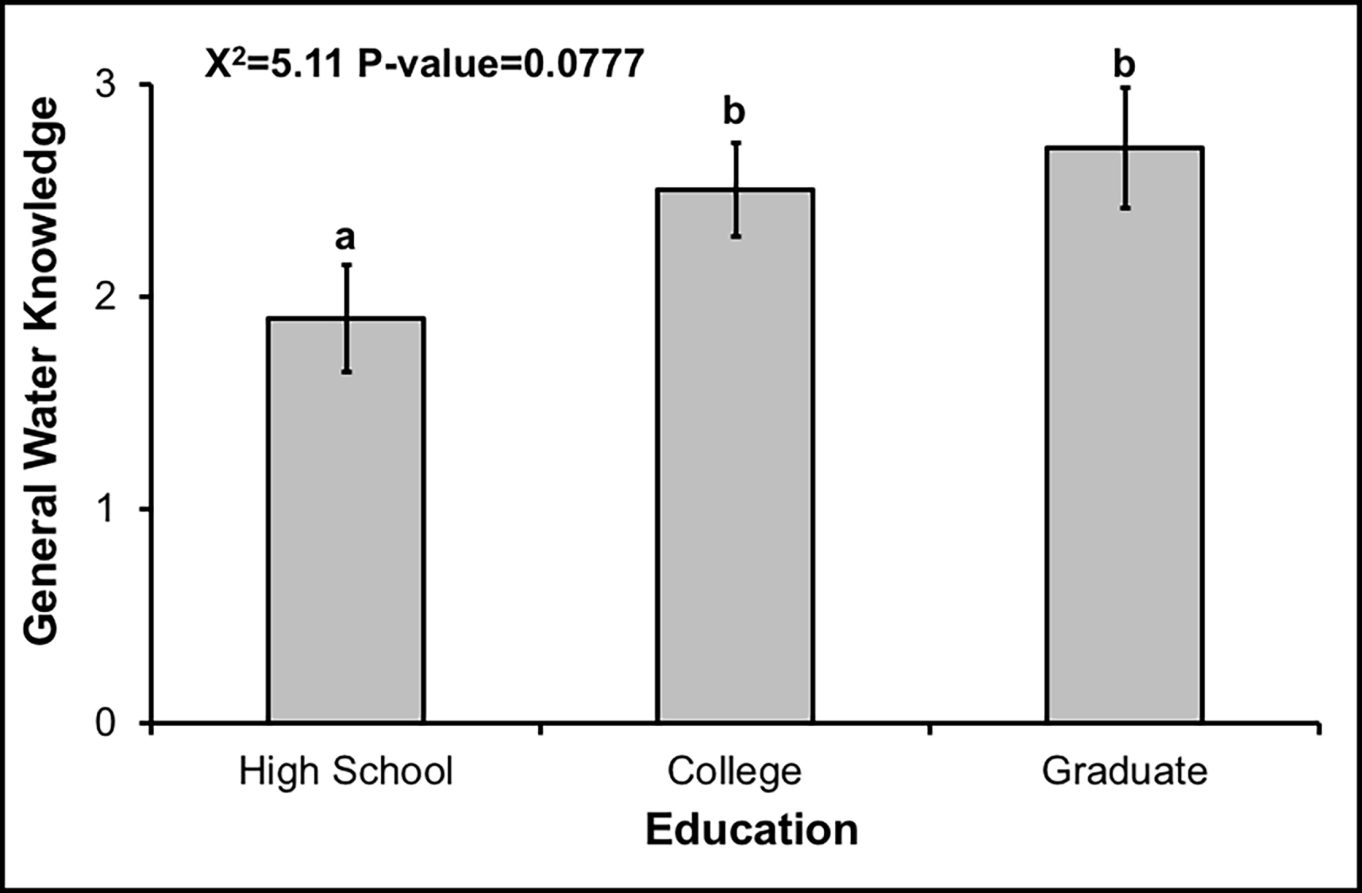
Greater knowledge by respondents with higher education. Mean (±1 SE) overall water resources knowledge scores by respondent education level. Respondents received overall water resources knowledge scores from 0–8 based on the number of correct answers they gave to eight questions. Different lowercase letters denote statistical significance among factor levels (p<0.05).

**Fig 3:**
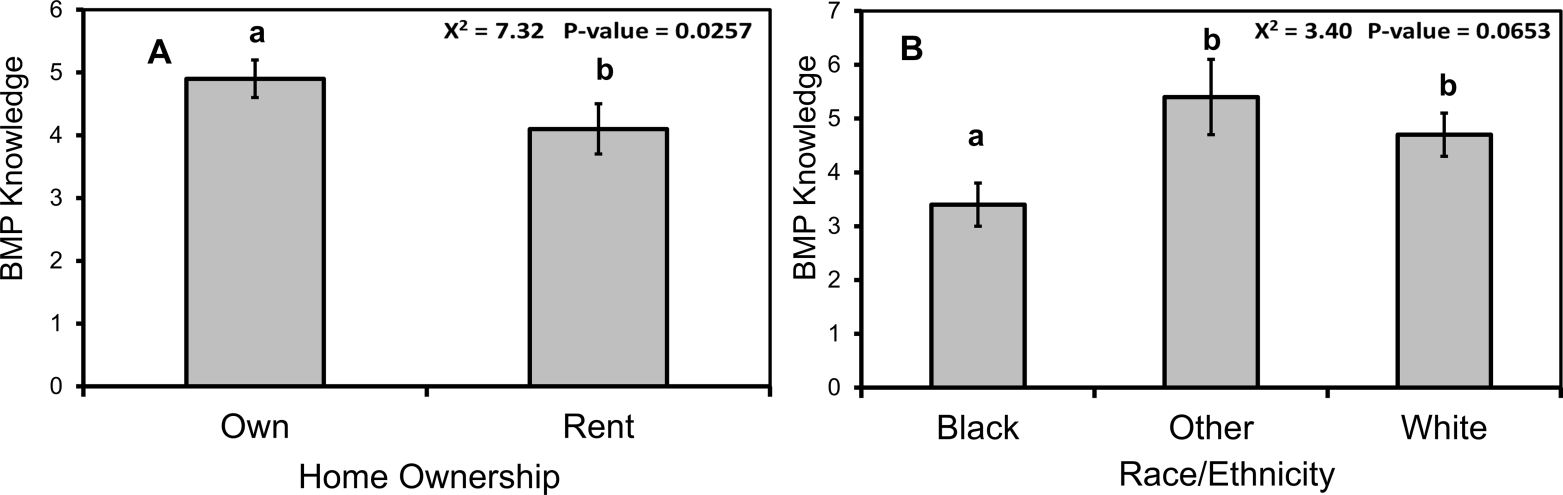
Greater BMP knowledge by respondents who identified as Caucasian or other races and who resided in owned dwellings. Mean (±1 SE) BMP knowledge scores by (A) respondent homeownership status and (B) race/ethnicity. Respondents received BMP knowledge scores based on the number of nine BMPs that they indicated familiarity with. Different lowercase letters denote statistical significance among factor levels (p<0.05).

Combined across both watersheds, mean overall and BMP knowledge scores were low on our 8 and 9-point scales, being 2.39 ± 1.51 and 4.20 ± 2.63, respectively. Moreover, for some knowledge questions, very few respondents gave correct answers. For example, only 27 out of 297 respondents correctly indicated that their county, city, town, or homeowner's association provides rebates for implementing BMPs. Overall water knowledge varied with almost all individual and household-level demographic factors, except individual gender, in single-factor tests (Table 1). In the final multi-factor model, however, overall water knowledge only varied marginally with education (χ^2^_1_ = 5.11, p = 0.0777), with respondents with college or graduate level education having greater knowledge than respondents with high-school education (Fig 2). Knowledge of BMPs varied by individual education and race, as well as watershed, household income, association membership and ownership status, in single-factor tests (Table 1). In multi-factor tests, BMP knowledge varied with individual race (χ^2^_1_ = 7.32, p = 0.0257) and marginally with household ownership status (χ^2^_1_ = 3.40, p = 0.0653), with respondents that are Caucasian or other races, and that were in owned dwellings, having greater BMP knowledge than respondents that were African American and who are renters, respectively (Fig 3).

**Table 1:** Linear model results testing respondent demographics on knowledge.

		Overall water knowledge		BMP knowledge	
Factor	df	X^2^	p ^a^	X^2^	p ^a^
**Individual level**					
education	2	23.41	<0.0001	25.39	<0.0001
gender	1	1.91	0.1672	3.34	0.0677
age	1	5.34	0.0209	1.75	0.1861
race	2	20.61	<0.0001	26.82	<0.0001
financial responsibility	1	3.41	0.0647	1.32	0.2513
**Household level**					
watershed	1	12.29	0.0005	17.18	<0.0001
income	1	6.24	0.0125	8.38	0.0038
children	1	6.36	0.0117	2.45	0.1177
association membership	1	10.03	0.0015	14.00	0.0002
house ownership	1	9.65	0.0019	13.90	0.0002

^a^ Factors with p<0.250 were included in multi-factor models.

### Attitudes

**Fig 4:**
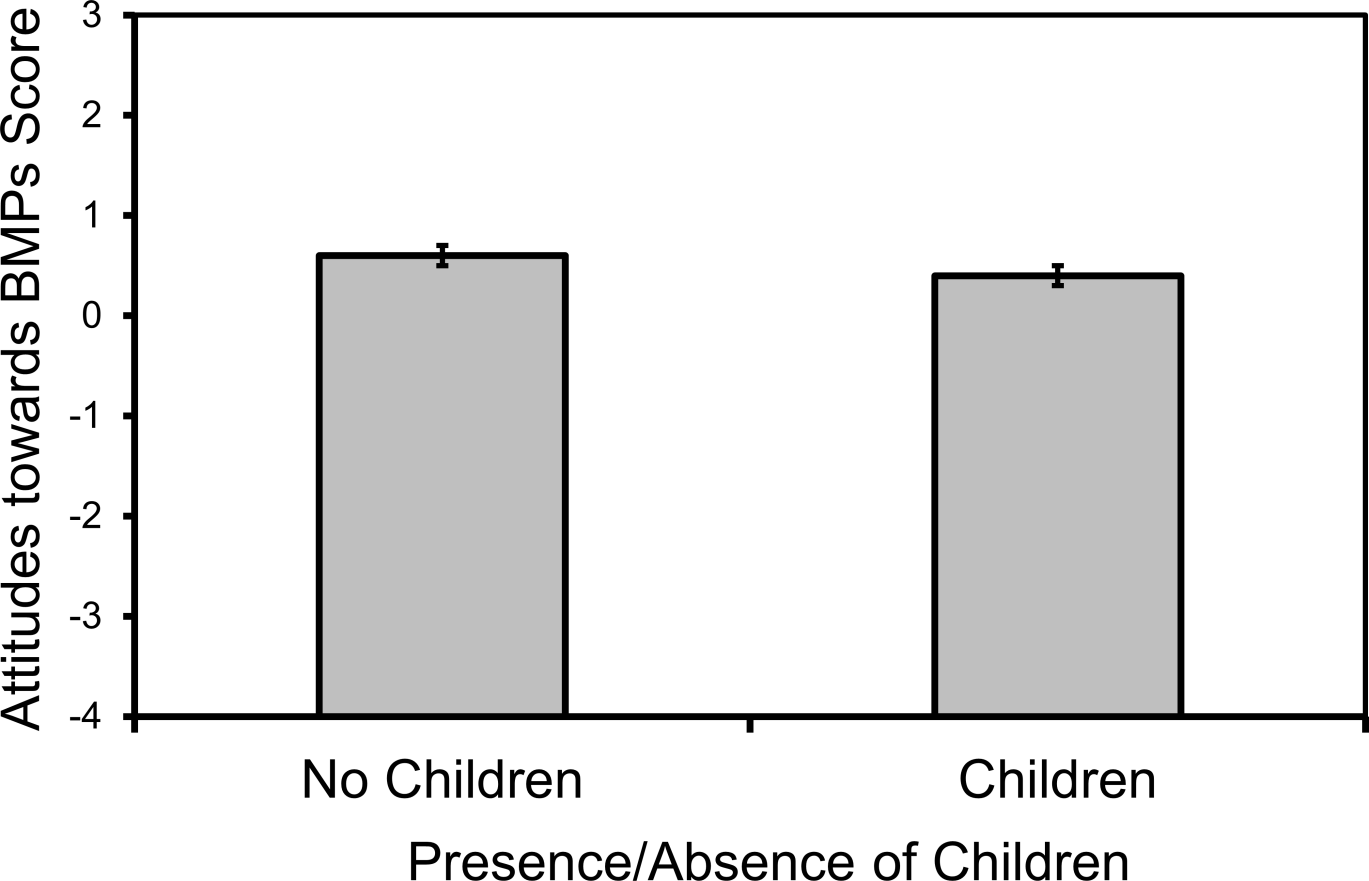
More favorable attitudes toward BMPs by respondents without children. Mean (±1 SE) attitudes towards BMPs by household presence of children. Respondents received a mean attitude score of 1–4 based on their agreement with six statements on a four-point as an overall index of their motivation to protect water resources.

**Fig 5:**
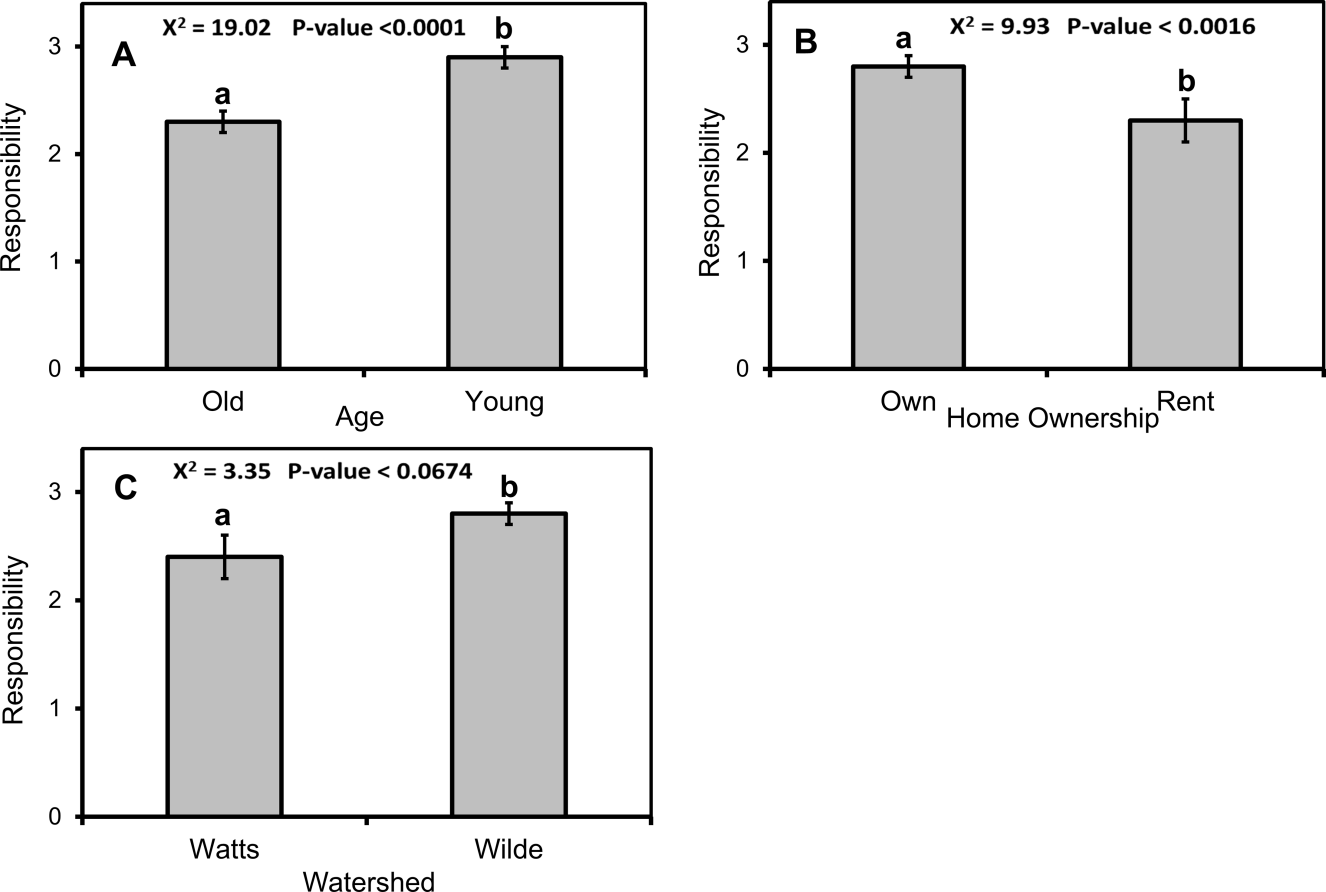
Respondent demographics predict the perceived responsibility for protecting Chesapeake Bay of individual households versus government. Mean (±1 SE) perceived responsibility for protecting Chesapeake Bay by respondent (A) age, (B) home-ownership status, and (C) watershed. Respondents were assessed on the degree to which they thought government versus individuals are responsible for stormwater runoff based on a 5-point Likert scale, with a larger number indicating greater responsibility for the individual. Different lowercase letters denote statistical significance among factor levels (p< 0.05).

Combined across both watersheds, the mean attitude score toward water resources was 3.00 ± 0.03, which corresponded to “agree” on our four-point scale to our six positive statements. Overall, the most positively perceived BMP was reducing fertilizer use, which has a mean attitude score eight times higher than the least liked BMP, lawn depression. Attitudes towards water resources only varied with household membership in a housing association, and marginally with watershed and household ownership status (Table 2). In multifactor tests, membership in a housing association remained in the final model but it was not significant (χ^2^_1_ = 1.01, p = 0.3138). Overall attitudes toward BMPs only marginally varied with the presence of children in a household (Table 2), with more favorable attitudes among respondents without children (Fig 4). No other variables had a P-value < 0.250. Respondents’ opinions of the roles of individuals versus government in protecting Chesapeake Bay varied with individual education, age, and race, watershed, and household ownership status (Table 2). In multi-factor tests, responsibility varied with respondent age (χ^2^_1_ = 19.02, p<0.0001) and household ownership status (χ^2^_1_ = 9.93, p<0.0016), and marginally with watershed (χ^2^_1_ = 3.35, p<0.0674). Respondents who were younger, that lived in owned dwellings, and that were from WL watershed thought individuals should have a larger role than government (Fig 5).

**Table 2:** Linear model results of respondent demographics on attitudes.

		Attitudes toward water resources		Attitudes toward BMPs		Responsibility	
Factor	df	X^2^	p ^a^	*X*^2^	p ^a^	X^2^	p ^a^
**Individual level**							
education	2	3.61	0.1641	0.31	0.8548	6.22	0.0447
gender	1	0.20	0.6520	0.08	0.7797	2.05	0.1521
age	1	0.01	0.9205	0.02	0.9021	8.84	0.0029
race	2	2.76	0.2521	0.61	0.7368	8.13	0.0171
financial responsibility	1	2.24	0.1346	0.23	0.6334	0.15	0.6944
**Household level**							
watershed	1	3.76	0.0526	0.29	0.5916	7.36	0.0067
income	1	1.33	0.2479	0.02	0.8854	1.60	0.2054
children	1	0.14	0.7058	3.53	0.0601	1.77	0.1832
association membership	1	4.55	0.0329	0.73	0.3914	0.61	0.4330
house ownership	1	3.78	0.0519	0.00	0.9777	7.79	0.0053

^a^ Factors with p<0.250 were included in multi-factor models.

### Practices

**Fig 6:**
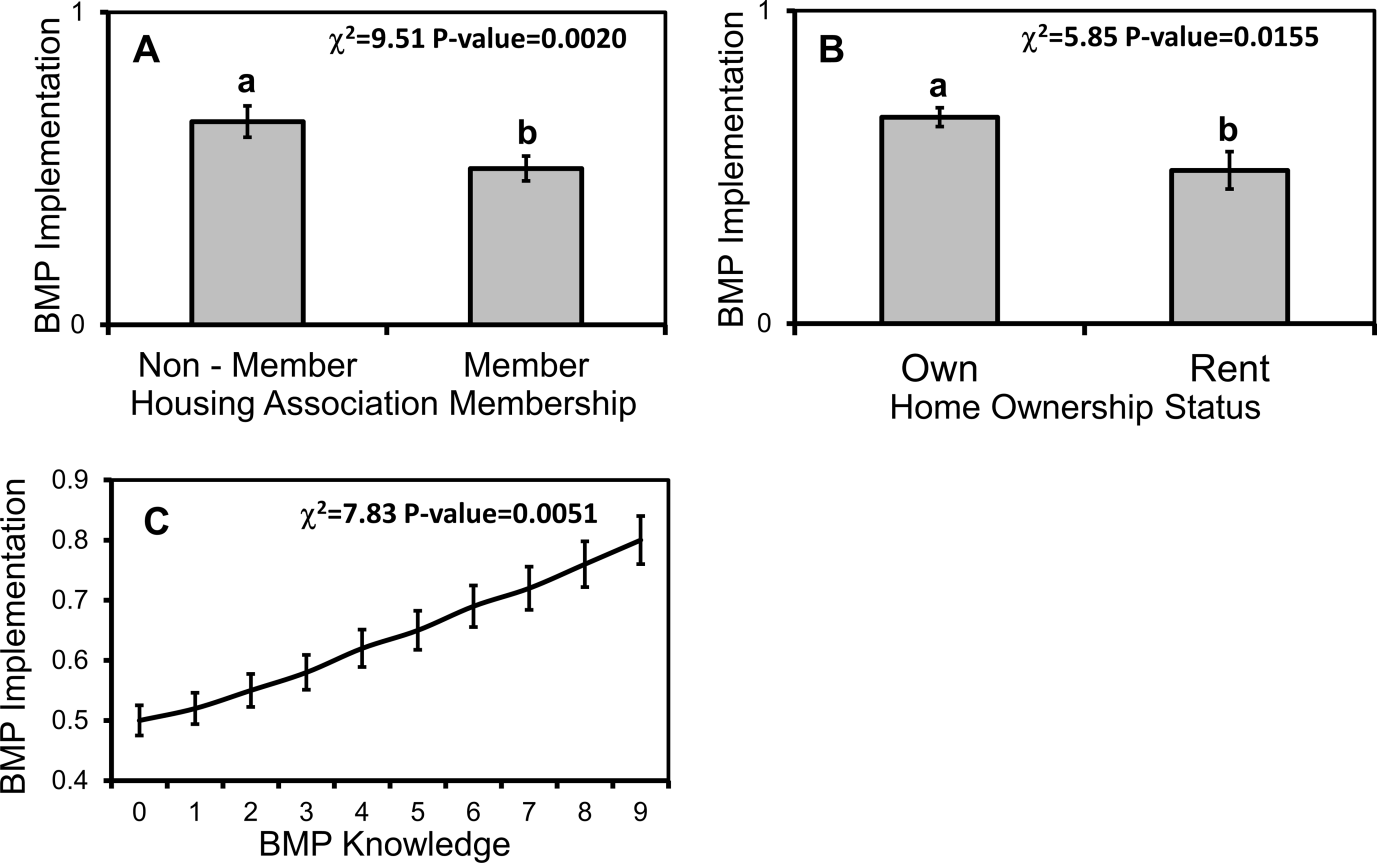
Respondent demographics and knowledge predicted BMP implementation. Mean (±1 SE) likelihood of BMP implementation by respondent (A) housing association membership, (B) homeownership status, and (C) BMP knowledge score. Respondents received BMP knowledge scores of 0–9 based on their reported familiarity with nine common BMPs. Different lowercase letters denote statistical significance among factor levels (p< 0.05).

Combined across both study watersheds, 63.3% (n = 188/297) of respondents reported practicing at least one BMP. The most common BMP was reducing fertilizer use (42.1%, 125/297), followed by downspout disconnection (36.0%, 107/297), and natural landscaping (25.4%, 76/297). Less than 10.0% of respondents reported implementing each of the other five BMPs that that were listed in the survey (Table 3). In single factor tests, the implementation of at least one BMP was strongly related to household ownership, and the respondent's financial responsibility, overall water knowledge, BMP knowledge, and attitudes towards water resources (Table 4). In multi-factor models, household ownership (χ^2^_1_ = 5.85, p = 0.0155) and individual BMP knowledge (χ^2^_1_ = 7.83, p = 0.0051) remained related to BMP implementation, while association membership also emerged as a strong predictor (χ^2^_1_ = 9.51, p = 0.0020). Individual financial responsibility (χ^2^_1_ = 3.55, p = 0.0594) and overall water knowledge (χ^2^_1_ = 3.26, p = 0.0710) were marginally significantly related to BMP implementation. Respondents who owned their home, who had greater knowledge, especially specific knowledge of BMPs, and who were not part of a housing association were more likely to live in a household that implemented a BMP (Fig 6).

**Table 3:** Percentage of respondents practicing Best Management Practices (BMPs).

	Watts Branch (n = 194) ^a^	Wilde Lake (n = 105) ^a^	Total (n = 299) ^a^
Reducing fertilizer	24.7 (48)	73.3 (77)	41.8 (125)
Downspout disconnection	40.7 (79)	26.7 (28)	35.8 (107)
Natural landscaping	21.6 (42)	32.4 (34)	25.4 (76)
Lawn infiltration	7.2 (14)	14.3 (15)	9.7 (29)
Pervious paving	9.8 (19)	8.6 (9)	9.4 (28)
Rain barrels	7.2 (14)	9.5 (10)	8.0 (24)
Lawn depression	3.6 (7)	6.7 (7)	4.7 (14)
Rain gardens	2.6 (5)	5.7 (6)	3.7 (11)

^a^ Respondent numbers in parentheses.

**Table 4:** Linear model results of respondent demographics, knowledge, and attitudes on BMP implementation.

		Any BMP		Specific BMP						Numbers of BMPs	
				Reducing fertilizer		Downspout disconnection		Natural landscaping			
Factor	df	X^2^	P ^a^	X^2^	p ^a^	X^2^	p ^a^	X^2^	p ^a^	X^2^	p ^a^
Individual-level demographics											
education	2	0.56	0.7568	1.92	0.3828	5.94	0.0512	1.32	0.5171	1.89	0.3882
gender	1	0.48	0.4878	0.20	0.6523	4.17	0.0412	1.91	0.1668	0.00	0.9686
age	1	3.76	0.0525	2.23	0.1355	0.13	0.7182	3.72	0.0537	1.87	0.1710
race	2	1.28	0.5263	1.55	0.4618	4.45	0.1079	7.61	0.0233	2.84	0.2417
financial responsibility	1	8.92	0.0028	13.52	0.0002	0.87	0.3512	5.90	0.0151	11.38	0.0007
Household-level demographics											
watershed	1	0.09	0.7620	1.31	0.2517	5.47	0.0193	4.46	0.0347	1.26	0.2619
income	1	0.24	0.6271	0.00	0.9527	4.80	0.0284	0.13	0.7175	0.12	0.7267
children	1	0.00	0.9946	0.14	0.7091	0.13	0.7222	1.47	0.2250	1.18	0.2772
association membership	1	1.97	0.1609	0.56	0.4523	2.28	0.1311	2.25	0.1334	0.26	0.6085
house ownership	1	13.11	0.0003	15.33	<0.0001	0.02	0.8907	16.19	<0.0001	16.13	<0.0001
Overall water knowledge	1	13.35	0.0003	15.99	<0.0001	2.86	0.0909	16.33	<0.0001	20.72	<0.0001
BMP knowledge	1	17.67	<0.0001	4.37	0.0365	5.79	0.0161	22.27	<0.0001	37.04	<0.0001
Specific BMP knowledge	1	-	-	4.46	0.0347	6.99	0.0082	33.87	<0.0001	-	-
Attitudes toward water resources	1	4.53	0.0333	3.76	0.0524	0.04	0.8336	8.98	0.0027	9.77	0.0018
Attitudes toward BMPs	1	0.15	0.6977	0.71	0.4009	0.12	0.7333	9.45	0.0021	5.71	0.0169
Attitude to specific BMP	1	-	-	8.66	0.0032	6.99	0.0082	21.21	<0.0001	-	-
Roles of individuals versus government	1	0.00	0.9521	1.30	0.2538	0.40	0.5260	3.76	0.0524	2.77	0.0962

^a^ Factors with p<0.250 were included in multi-factor models.

**Fig 7:**
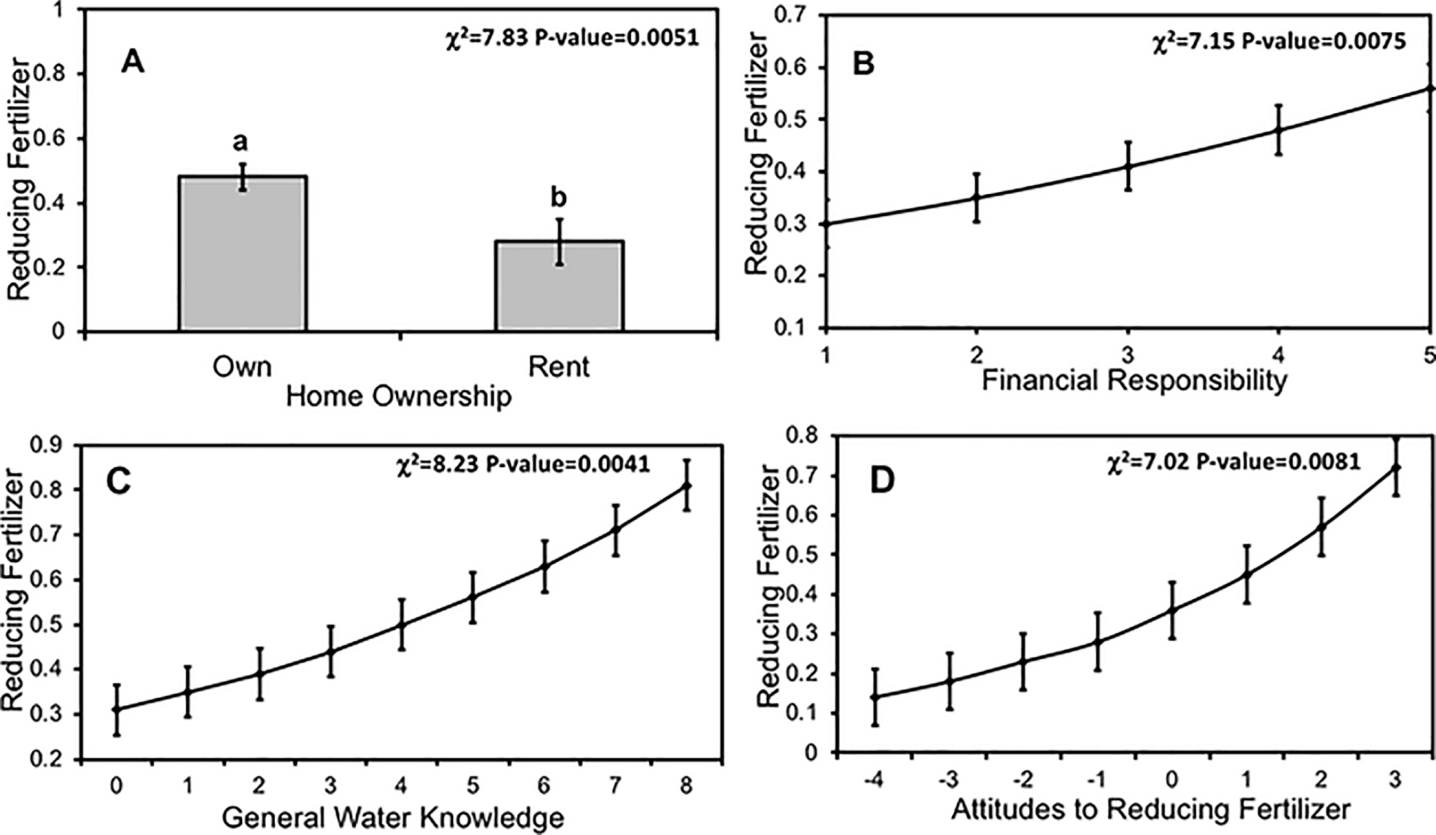
Respondent demographics, knowledge, and attitudes predicted likelihood of reducing fertilizer. Mean (±1 SE) likelihood of reducing fertilizer by respondent (A) homeownership status, (B) financial responsibility within the household, (C) overall water resources knowledge score, and (D) attitudes towards reducing fertilizer. Respondents received overall water resources knowledge scores of 0–8 based on the number of correct answers they gave to eight questions. Different lowercase letters denote statistical significance among factor levels (p< 0.05).

The most common BMP, reducing fertilizer, was related to a respondent's financial responsibility, overall water knowledge, BMP knowledge, specific knowledge of and attitude to reducing fertilizer, and to household ownership (Table 4). In multi-factor models, household ownership, household financial responsibility, overall water knowledge, and attitudes toward reducing fertilizer were related to reduced fertilizer use. Respondents who owned their home, who had a larger role in household financial decisions, who had greater overall water knowledge of water resources, and who had more favorable attitudes toward reducing fertilizer were more likely to reduce fertilizer (Fig 7).

**Fig 8:**
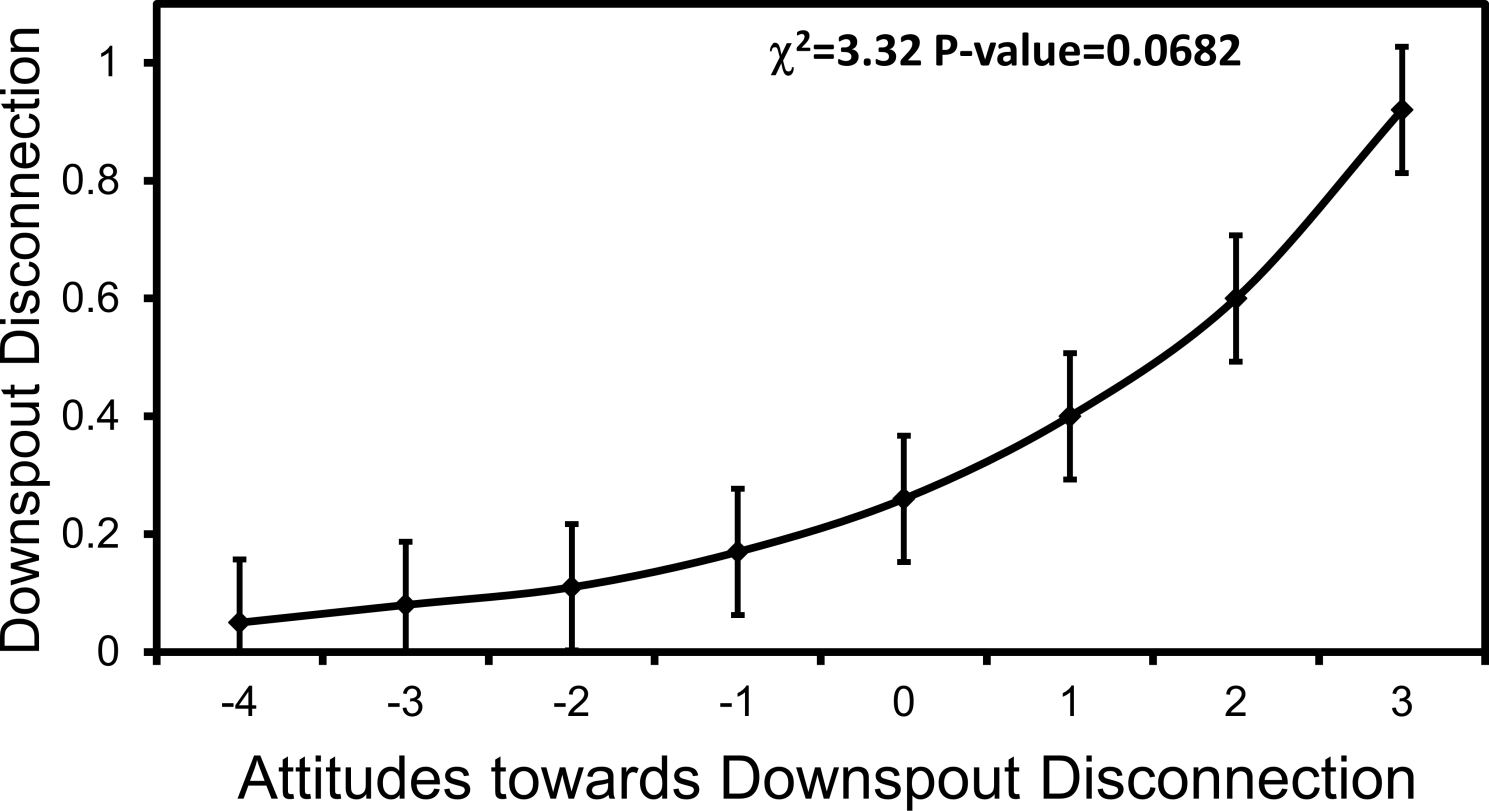
Favorable respondent attitudes toward downspout disconnections predict increased likelihood of their implementation. Mean (±1 SE) likelihood of implementing downspout disconnections by respondent attitudes toward the BMP. Respondents received attitude scores of -4-3 based on their agreement with negative and positive statements toward downspout disconnections. Different lowercase letters denote statistical significance among factor levels (p<0.05).

The implementation of downspout disconnections was related to a respondent's education, gender, watershed, household income, overall water knowledge of BMPs, and specific knowledge and attitudes toward downspout disconnections (Table 4). In multi-factor models, only education remained an important predictor (χ^2^_2_ = 6.93, p = 0.0313), but no pairwise contrasts were significantly different (P > 0.05; data not shown). Overall water knowledge emerged as only marginally related (χ^2^_2_ = 3.69, p = 0.0549). When models included attitudes toward this specific BMP and run on the subset of respondents that reported being familiar with it, education and overall water knowledge ceased to be related to the implementation of downspout disconnections and only attitudes toward the BMP emerged as being marginally significant (χ^2^_2_ = 3.32, p = 0.0682), with a trend indicating that more favorable attitudes promoted greater implementation (Fig 8).

**Fig 9:**
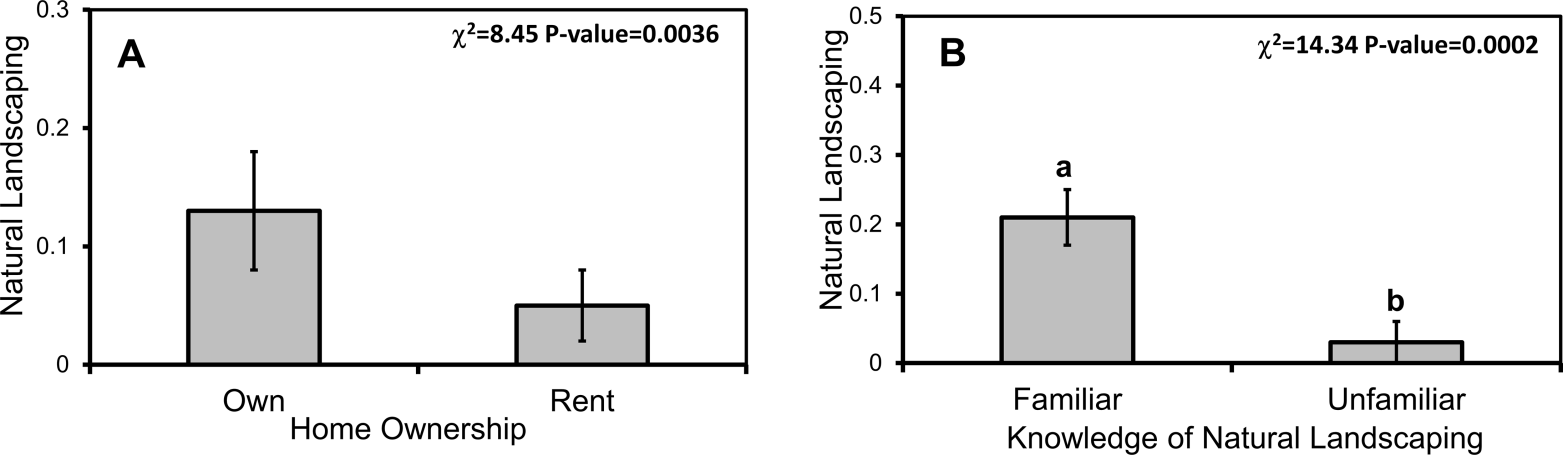
Home ownership and greater BMP knowledge predict greater practice of natural landscaping. Mean (±1 SE) implementation of natural landscaping by (A) homeownership status and (B) BMP knowledge. Respondents received BMP knowledge scores of 0–9 based on their reported familiarity with nine common BMPs. Different lowercase letters denote statistical significance among factor levels (p<0.05).

**Fig 10:**
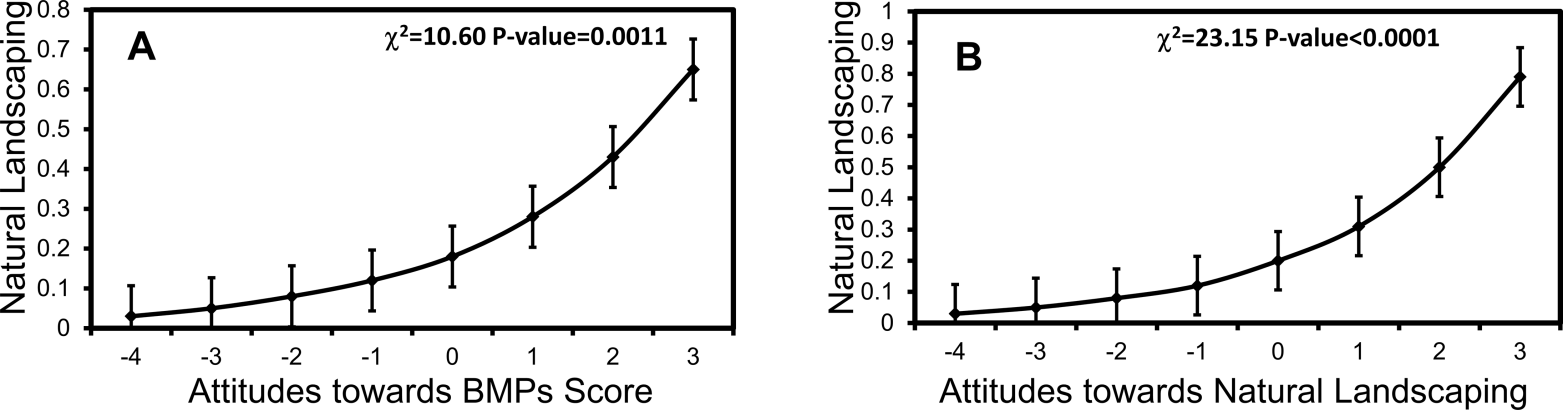
Favorable respondent attitudes toward BMPs in general and natural landscaping in particular predict increased likelihood of natural landscaping practice. Mean (±1 SE) implementation of natural landscaping by respondent (A) attitudes to BMPs in general and (B) to natural landscaping in particular. Respondents received attitude scores of -4-3 based on their agreement with negative and positive statements towards natural landscaping and eight other BMPs. Different lowercase letters denote statistical significance among factor levels (p<0.05).

Natural landscaping was related to a respondent's household financial responsibility, watershed, ownership status, and knowledge and attitudes toward water resources, BMPs, and natural landscaping in particular (Table 4). In multi-factor models, household ownership (χ^2^_2_ = 8.45, p = 0.0036), overall attitudes toward BMPs (χ^2^_2_ = 10.60, p = 0.0011), and specific knowledge (χ^2^_2_ = 14.34, p = 0.0002) and specific attitudes (χ^2^_2_ = 23.15, p<0.0001) towards natural landscaping were all related to natural landscaping practice. Respondents that were owners, had greater BMP knowledge, more favorable attitudes toward BMPs, and who had favorable attitudes toward natural landscaping in particular were all more likely to practice the BMP (Figs 9 and 10).

**Fig 11:**
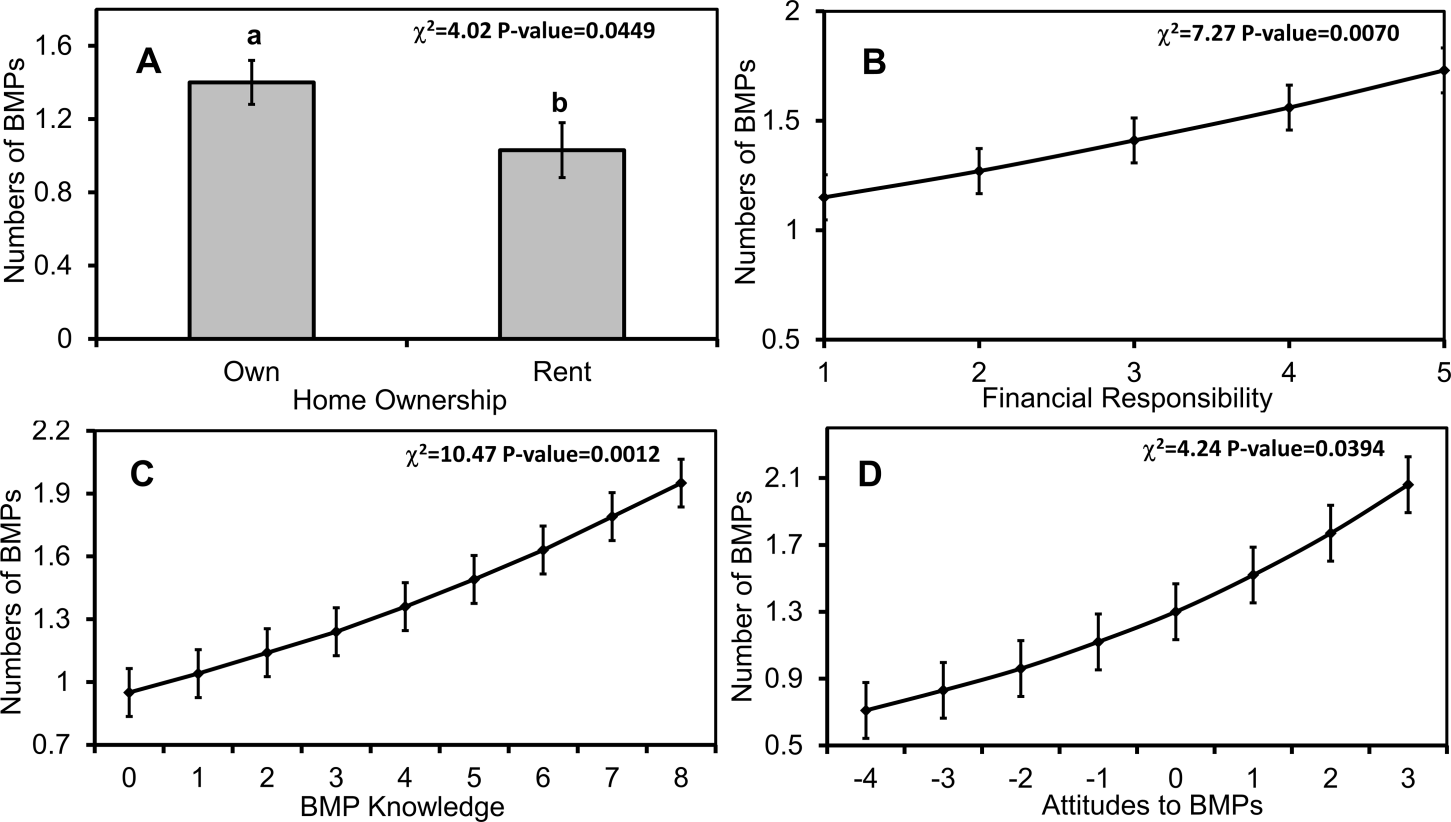
Number of implemented BMPs as predicted by demographics, knowledge, and attitudes. Mean (±1 SE) numbers of implemented BMPs by (A) homeownership status, (B) respondent financial responsibility, (C) BMP knowledge, and (D) attitudes toward BMPs. Different letters denote statistical significance among factor levels (p<0.05). Respondents received BMP knowledge scores of 0–9 based on their reported familiarity with nine common BMPs, and mean attitude scores from -4-3 based on their agreement with negative and positive statements of nine BMPs. Different lowercase letters denote statistical significance among factor levels (p<0.05).

In single factor tests, total household BMP numbers varied with household ownership, the degree of a respondent's responsibility to household finances, overall water knowledge, BMP knowledge, attitudes toward water resources, attitudes toward BMPs, and marginally with attitudes on the roles of individuals versus governments (Table 4). In multi-factor models, BMP numbers varied with financial responsibility (χ^2^_1_ = 7.27, p = 0.0070), BMP knowledge (χ^2^_1_ = 10.47, p = 0.0012), attitudes toward BMPs (χ^2^_1_ = 4.24, p = 0.0394), and household ownership (χ^2^_5_ = 4.02, p = 0.0449). Respondents who owned their own home, had a larger household financial responsibility, and who had greater knowledge of, and more favorable attitudes toward, BMPs were more likely to have higher numbers of BMPs (Fig 11).

### Specific incentives, barriers and education

When looking at respondent lifestyle preferences, the majority of respondents stated that they liked to garden (56.7%, 171/297), while a large proportion reported enjoying crabbing and fishing (40.4%, 120/297), and identifying as an environmentalist (45.8%, 136/272). Only 4 respondents indicated that they were a member of their local watershed organization (1.3%, n = 297). When looking at respondent concerns, the majority of respondents were concerned about the overall health of the Chesapeake Bay (80.8%, 240/297), mosquito breeding in BMPs (78.5%, 233/297), and a large proportion were also concerned about safety issues related to stormwater BMPs (40.7%, 121/297). Chi-square tests of association between the implementation of each of the three most common BMPs with lifestyle preferences and concerns found that those who were concerned about the health of Chesapeake Bay were more likely to implement reduced fertilizer use (47.8%, 113/240 vs. 21.1%, 12/57; χ^2^_1_ = 12.80, p = 0.0003), those who volunteered at environmental events (53.6%, 30/56 vs. 32.0%, 77/164; χ^2^_1_ = 9.22, p = 0.0024) and who were concerned about safety issues (47.8%, 58/121 vs. 27.8%, 49/176; χ^2^_1_ = 12.56, p = 0.0004) were more likely to implement downspout disconnections, and those who considered themselves environmentalists were more likely to implement natural landscaping (34.6%, 47/136 vs. 18.0%, 29/161; χ^2^_1_ = 10.60, p = 0.0011). When analyses were re-run with respondents who owned their home and therefore likely had fewer restrictions implementing BMPs, we found that respondents who volunteered at environmental events (69.1%, 29/42 vs. 43.6%, 78/101; χ^2^_1_ = 8.84, p = 0.0030) and who were concerned about the health of Chesapeake Bay (53.3%, 98/184 vs. 24.32%, 9/37; χ^2^_1_ = 10.32, p = 0.0013) were more likely to reduce fertilizer use, and who volunteered at environmental events (59.5%, 25/42 vs. 30.7%, 55/179; χ^2^_1_ = 12.22, p = 0.0005) and who considered themselves an environmentalist (46.7%, 50/107 vs. 26.3, 30/114; χ^2^_1_ = 9.96, p = 0.0016) were more likely to reduce fertilizer use. All other associations were not significant after Bonferroni correction (p-values = 0.0037–0.9783).

**Fig 12:**
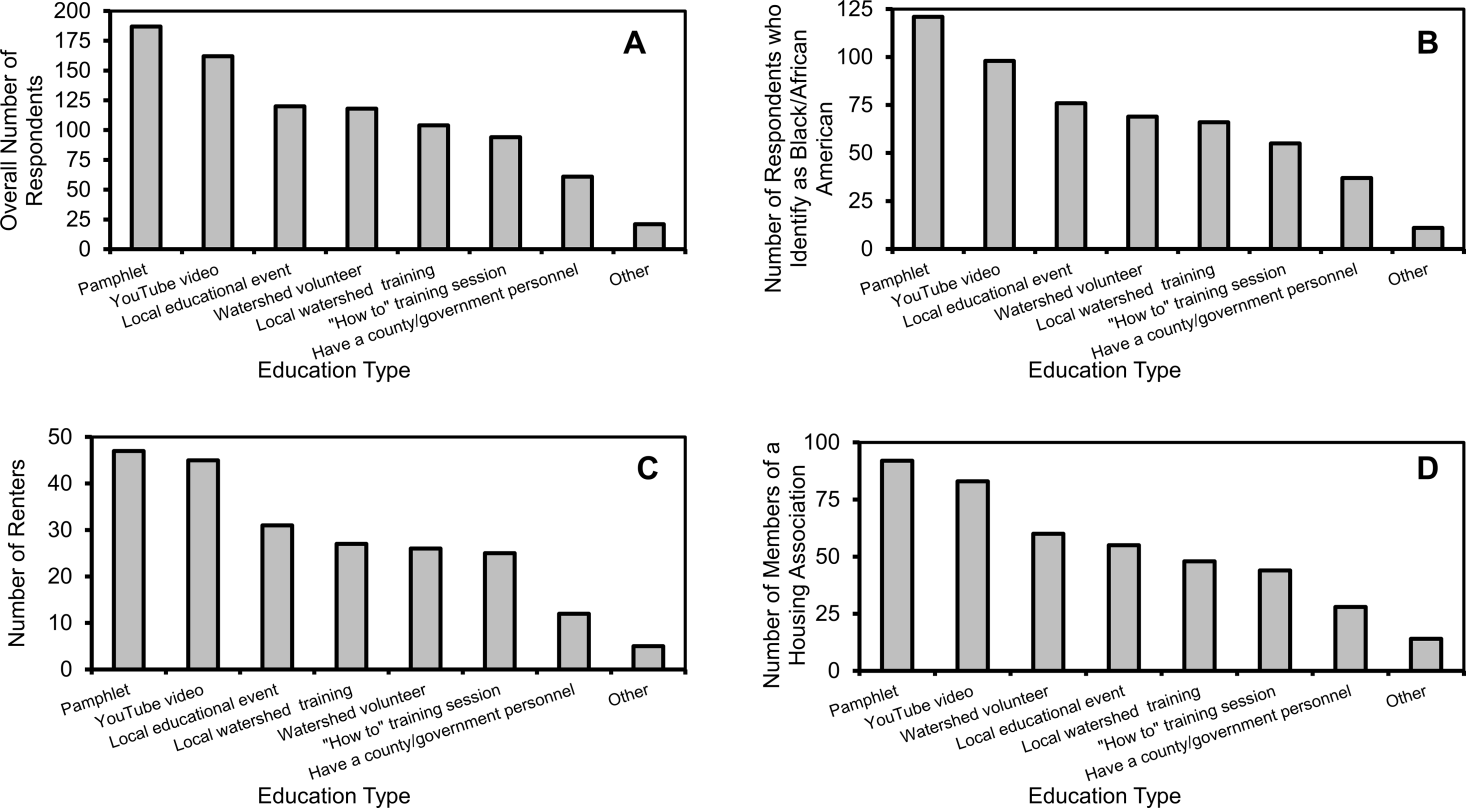
Education preferences as predicted by demographics. Numbers of respondents that identified specific education media as preferred by (A) total respondents, (B) respondents who identify as black/African American (C) renters, and (D) members of a homeowner’s association.

When looking at preferred educational/outreach methods, pamphlets were the preferred educational method of respondents, followed by YouTube videos (Fig 12A). When looking specifically at those demographic groups with lower knowledge of water resources or BMPs or groups with directly lower BMP implementation, there were similar findings. Those who identified as Black/African American, who had lower water resource and BMP knowledge, preferred pamphlets, followed by YouTube videos and local educational events (Fig 12B). Renters, who had lower BMP implementation, also preferred pamphlets, YouTube videos, and local educational events (Fig 12C). Finally, those who were members of a housing association, who also had lower BMP implementation, preferred pamphlets, YouTube videos, and lastly being visited by a watershed volunteer (Fig 12D).

## Discussion

There is a growing realization among water quality experts that more substantial reductions in NPS pollution and resultant improvements in watershed quality need community-based citizen engagement [7, 43]. This is especially the case in watersheds where numerous residential parcels constitute a large proportion of the total land cover on which household BMPs could be implemented. However, few studies have rigorously examined important social predictors of household BMP implementation [14, 29]. This study used data from a detailed questionnaire to empirically test relationships between knowledge, attitudes, and BMP implementation across socioeconomic and other gradients in the United States, and represents one of the few studies to rigorously examine potential social barriers to water quality management at the household level.

Among the most important findings of this study were the frequent and strong predictive relationships of resident knowledge on BMP implementation. Respondents with greater BMP knowledge (or familiarity) were more likely to reside in households that practice at least one BMP, practice more BMPs, and practice the most common BMP, fertilizer reduction. Implementation of natural landscaping was also positively predicted by knowledge of that BMP. The questionnaire in this study cited common BMPs on which there is considerable evidence for their effectiveness in reducing NPS pollution [43, 44]. Therefore, our findings suggest that a lack of familiarity with these BMPs in general and of some BMPs in particular is likely a strong barrier to better water quality in many residential watersheds. Few prior studies have examined the effect of knowledge on residential BMP adoption in urban areas, however one precedent study by Brehm et al. 2013 [29] reached a similar conclusion to our study here, that knowledge of BMPs was an important factor predicting BMP implementation among residents. These findings are also consistent with conclusions made by Cottrell and Graefe 1997 [45] that knowledge was a significant factor in predicting the implementation of environmentally responsible practices.

Importantly, this study found that variation in BMP knowledge was explained by specific demographic factors. Respondents who defined themselves as Caucasian or other races (not Caucasian or African American in this study) had higher mean BMP knowledge than respondents that identified as African American, as did respondents that lived in owned dwellings compared to renters. These findings indicate that, through important variation in resident knowledge, there is a clear connection between the socioeconomic and cultural environment and BMP implementation, and by extension water quality. These findings are broadly consistent with a growing body of literature demonstrating that environmental and natural resource management is heavily influenced by a society’s socioeconomic and cultural context [46–48]. For example, a study in New Jersey found that Asian Americans and Spanish-language Hispanic Americans had less concern of environmental pollution than other racial/ethnic groups [49]. Despite these clear trends, we caution strictly applying described relationships between environmental practices and race to other systems. Race is a socially constructed variable and therefore is complex and dynamic, varying by factors such as location, culture, and language. It can also interact with socioeconomic status in subtle ways not detected in our study here. For example, Maslow’s hierarchy-of-needs theory states that individuals are concerned about meeting physiological and safety needs before other types of needs, including for example those environmental practices [50], which may not be adequately explored in a KAP questionnaire.

Respondent attitudes also predicted BMP implementation in this study, although not as frequently as that of knowledge. Households with more positive attitudes toward BMPs tended to implement more BMPs, and respondents with more positive attitudes toward reducing fertilizer and natural landscaping in particular, were more likely to implement those specific BMPs. Interestingly however, respondent attitudes toward BMPs were not explained by most demographic factors, with the exception being that of the presence of children. Respondents in households with children were more likely to have less favorable attitudes toward BMPs. One explanation for this result may be that care-givers of children have higher safety concerns of water-holding stormwater structures. However, follow-up test reveals that although nearly half of total respondents expressed safety concerns of stormwater structures (40.7%, 121/297), there was no association between safety concern and the presence of children in the household (*X*^2^ = 0.0056, p = 0.9395). Although we found that respondents had favorable overall attitudes toward water resources, variation in their level of favorability was not explained by any demographic factors nor did it predict BMP implementation. Interestingly, respondent perceptions on whether government or individuals were mainly responsible for protecting Chesapeake Bay were related to demographic factors. Residents who were younger, that lived in owned dwellings, and that were from WL watershed thought that individuals should take greater responsibility. This finding is broadly consistent with a previous study that found that resident perceptions of the role of individuals and governments in environmental protection differed by race/ethnicity [49]. Nevertheless, again, this aspect of resident attitudes did not predict BMP implementation.

It is also possible that the implementation of any particular BMP may not be a conscious choice of the household but rather a result of always practicing it. For example, if a residence already had natural landscaping or pervious paving when the household acquired it, then a respondent might have reported using the BMP without taking any conscious action to implement it. The questionnaire in this study requested respondents to report BMPs that they already installed or that the respondent used. For some BMPs, including the most commonly reported, reducing fertilizer, downspout disconnections, and natural landscaping, there are potential immediate benefits for not continuing their practice (e.g., increased growth and/or perceived attractiveness of their yard) that might encourage their discontinuation by the household. Further, this study assumed that knowledge and attitudes precede implementation. It is possible that that these variables are instead influenced by BMP implementation. For example, a resident may know little about water resources or a specific BMP but learned about the BMP and water resources after acquiring a residence with a BMP or after a BMP is implemented by a landlord.

Although KAP questionnaires provide a powerful approach to characterize resident demographics, knowledge, and behaviors, as well as describe their inter-relationships, they cannot determine the cognitive decision-making processes that predict BMP implementation or continued use, which are likely to be complex and variable among both respondents and BMPs. More qualitative research methods, such as focus groups or interviews, may be more effective at exploring subtler and often complex interactions. These may include the role attitudes may play in BMP implementation, between BMP implementation with other needs along socioeconomic gradients, and the influence that pre-existing BMPs may have on the knowledge and attitudes of household residents. Follow-up focus group interviews after questionnaires have been implemented successfully by other studies (e.g., Randolph and Troy 2008 [49]) that have explore at behavioral trends [51].

The predictive relationships on BMP implementation of resident knowledge and attitudes in this study broadly supports the information-deficit hypothesis of environmental education [52]. This hypothesis suggests that public skepticism or hostility to science, technology, or more specifically, to environmental conservation, is a result of a lack of information [53–55]. Study respondents generally agreed with questionnaire statements that represented positive associations with, and utilization of, local and regional water resources, and a perceived ability to help restore Chesapeake Bay. However, combined across both watersheds, resident knowledge of water resources and of BMPs were low on the scales that were used in the questionnaire. Although correctly answering our knowledge questions does not indicate that a resident has reached a required knowledge level to appropriately manage water resources, low scores indicate that there is likely considerable scope for improving the knowledge of residents towards water resources and their management.

Pamphlets and YouTube videos were the two most preferred types of education delivery across all respondents, as well as respondents who identified as African American, who were renters, or who were members of a housing association; three demographic groups that either had lower rates of BMP implementation or knowledge compared to comparison groups (see above). Identifying preferred outreach approaches of target populations is important to tailor education programs. This may be especially important because only 4 out of 297 respondents reported being a member of a local watershed organization where they might be exposed to regular education on stormwater management and broader environmental messages. Our study showed that residents who agreed with various statements that reflected environmental lifestyle preferences and concerns were more likely to implement the most common BMPs. Interestingly, pamphlets and YouTube videos are “passive” in that they do not involve an experimental learning activity or face-to-face interaction with an education communicator. A large body of research has indicated that passive education tends to be less effective at achieving behavior change generally and in environmental management in particular [56, 57]. Respondent’s preference for passive education in this study may reflect prior adverse interactions with people with regards to stormwater management or a perception that they may be pressured into purchasing a BMP. Further research, needs to better understand the underlying factors dictating resident perceptions of education approaches.

In addition to predicting BMP implementation indirectly via knowledge, some demographic factors directly predicted variation in BMP implementation. Households that owned their dwellings were more likely to practice at least one BMP, practice both reduced fertilizer and natural landscaping, and practice more BMPs. These findings are consistent with considerable environmental management literature that indicates that citizens are more likely to invest in their environment if they have an economic and emotional involvement in it [58–60]. For example, Blake 1999 [60] found that those who did not own their homes did not implement environmental practices because they would not directly benefit from these actions. More practically, even if renters want to implement a BMP, they have restrictions to doing so by their landlord. In our study, there were negative associations between household membership in a housing association and BMP implementation. Similar to renters, members of housing associations may have additional restrictions to BMP implementations, including additional permitting by the housing association and associated restrictions on building materials etc. that may act as an additional barrier to implementation. This study only surveyed single-family detached/stand-alone residences that had yards in which BMPs could be implemented. Households that live in multiple-unit dwellings (e.g., apartment complexes) would likely have more difficulty implementing BMPs even if they owned their unit.

## Conclusions

This study has identified key social predictors of BMP implementation that can be addressed in future education and outreach efforts to improve stormwater management. Residents lacking overall water knowledge of water resources and of BMPs may need to be more intensively studied using other instruments, including focus groups or interviews, to determine the most effective education approaches. Increasing BMP implementation of demographic groups that exhibit lower likelihood of BMP implementation independent of knowledge, such as renters and members of housing associations, may require changes in regulations to increase home ownership and require housing associations to allow residents to implement stormwater management on their properties.

## Supporting information

S1 Dataset Data compiled from the study questionnaire. (XLSX)
